# Neuroprotective effects of ceftriaxone after severe traumatic brain injury in male rats: a behavioral, biochemical, and histological study

**DOI:** 10.1097/MS9.0000000000004376

**Published:** 2025-11-25

**Authors:** Amirhossein Esfahani, Ali Siahposht-Khachaki, Fereshteh Talebpour Amiri, Tohid Emami Meybodi, Mobina Gheibi, Erfan Ghadirzadeh, Morteza Biabani, Ghazal Taghipour, Asal Safarbalou, Davood Farzin

**Affiliations:** a School of Medicine, Ramsar Campus, Mazandaran University of Medical Sciences, Ramsar, Iran; b Deparment of Physiology, Immunogenetics Research Center, Mazandaran University of Medical Sciences, Sari, Iran; c Psychiatry and Behavioral Sciences Research Center, Addiction Institute, Mazandaran University of Medical Sciences, Sari, Iram.; d Department of Anatomy, Molecular and Cell Biology Research Center, Mazandaran University of Medical Sciences, Sari, Iran; e Neuroscience Research Center, Iran University of Medical Sciences, Tehran, Iran; f Functional Neurosurgery Research Center, Research Institute of Functional Neurosurgery, Shohada Tajrish Neurosurgical Center of Excellence, Shahid Beheshti University of Medical Sciences, Tehran, Iran; g Department of Medical Laboratory Sciences, Razi Hospital, Mazandaran University of Medical Sciences, Sari, Iran; h Student Research Committee, School of Allied Medical Sciences, Mazandaran University of Medical Sciences, Sari, Iran; i Non-Communicable Diseases Institute, Mazandaran University of Medical Sciences, Sari, Iran; j Student Research Committee, School of Medicine, Mazandaran University of Medical Sciences, Sari, Iran; k Department of Pharmacology Sciences, School of Pharmacy, Mazandaran University of Medical Sciences, Ramsar, Iran; l Department of Pharmacology, School of Medicine, Mazandaran University of Medical Sciences, Sari, Iran

**Keywords:** beta-lactam, ceftriaxone, neuroprotection, rat, traumatic brain injury

## Abstract

**Background:**

Only a limited number of animal studies have demonstrated the neuroprotective effects of single dose of ceftriaxone (CTX) in traumatic brain injury (TBI). Consequently, the present study seeks to fill these research gaps by examining the impact of CTX on neurological and motor performance, brain edema, and blood–brain barrier (BBB) permeability in a rat model of severe diffuse TBI.

**Methods:**

Ninety-eight male Albino Wistar rats were subjected to TBI using the Marmarou method. The rats were divided into seven groups, each consisting of 14 animals: Intact, Sham, TBI, Vehicle (TBI + saline placebo injection), and three groups receiving single intraperitoneal (IP) injections of CTX at doses of 100, 200, and 400 mg/kg. Post-trauma assessments included measurements of the brain water content (BWC), BBB permeability, Veterinary Coma Scale (VCS), beam-walk (BW), beam-balance (BB), IL-10, IL-1β, TNF-α, and INF-γ concentrations, liver and kidney function tests, and histopathological analysis.

**Results:**

Administration of CTX at doses of 100 and 200 mg/kg resulted in significantly reduced BWC, enhanced BBB integrity, improved VCS scores, better performance on BW and BB tests, reduced IL-1β, TNF-α, and INF-γ, and increased IL-10, along with favorable histopathological outcomes and no signs of systemic toxicity. However, increasing the dose to 400 mg/kg did not yield further improvement.

**Conclusion:**

Our results demonstrate that CTX administration at doses of 100 and 200 mg/kg significantly improved neurological and motor function, reduced cerebral edema, enhanced blood-brain barrier integrity, and favorably modulated the post-traumatic inflammatory response. In contrast, the 400 mg/kg dose conferred no additional benefit.

## Introduction

Traumatic brain injury (TBI) refers to brain dysfunction caused by an external mechanical force such as a blow or jolt to the head, which can lead to temporary or permanent impairments in cognitive, physical, and psychosocial functions[1]. TBI symptoms can vary widely but often include headaches, dizziness, confusion, and changes in cognitive function[2]. TBI is a major cause of mortality and long-term impairment globally. Annually, approximately 64–74 million people suffer from TBI[3].

TBI initiates a complex series of pathological processes involving multiple cellular and molecular pathways[4]. TBI can be categorized into two phases: primary injury, which occurs at the moment of impact and includes conditions such as brain contusion, diffuse axonal injury, cerebral edema, and intracranial hemorrhage, and secondary injury, which involves a complex cascade of biochemical and cellular events that exacerbate the initial damage over days to months^[5,6]^. These secondary mechanisms include excitotoxicity, oxidative stress, inflammation, and neuronal cell death, which collectively contribute to long-term neurological deficits and poor outcomes^[7–9]^.

Excitotoxicity, which is driven by the excessive release of glutamate, is a critical component of secondary brain injury. Glutamate, the primary excitatory neurotransmitter in the central nervous system, accumulates in the extracellular space following TBI owing to both increased release from damaged neurons and impaired reuptake by astrocytes^[10,11]^. This excess glutamate overactivates postsynaptic glutamate receptors, leading to calcium overload, mitochondrial dysfunction, and neuronal death^[12,13]^. Among glutamate transporters, glutamate transporter-1 (GLT-1) is responsible for the majority of glutamate clearance in the brain, thereby preventing excitotoxicity. However, following TBI, GLT-1 expression is significantly downregulated, further exacerbating glutamate-mediated neuronal damage^[14–16]^.

Therapeutic strategies such as treatment with ceftriaxone (CTX), a beta-lactam antibiotic that crosses the blood-brain barrier, have shown promise for TBI-induced consequences^[17,18]^. Beyond its antimicrobial properties, CTX has been shown to upregulate GLT-1 expression, thereby enhancing glutamate clearance and reducing excitotoxicity^[18,19]^. It may also modulate autophagy pathway proteins that are upregulated after TBI, suggesting additional neuroprotective effects^[6,20,21]^.

Despite these promising findings, there are only a few studies regarding the neuroprotective effects of CTX in severe TBI models, especially its effect on cerebrospinal fluid (CSF) interleukins. Thus, the neuroprotective efficacy of CTX remains debatable, particularly in terms of behavioral outcomes and biochemical and histological changes[22]. Therefore, this study aimed to investigate the effects of IP CTX administration on neurological scores, motor function, cerebral edema, blood–brain barrier (BBB) permeability, and cytokine levels in CSF following severe TBI, providing further insights into its potential therapeutic role.

## Methods

**Figure 1.**
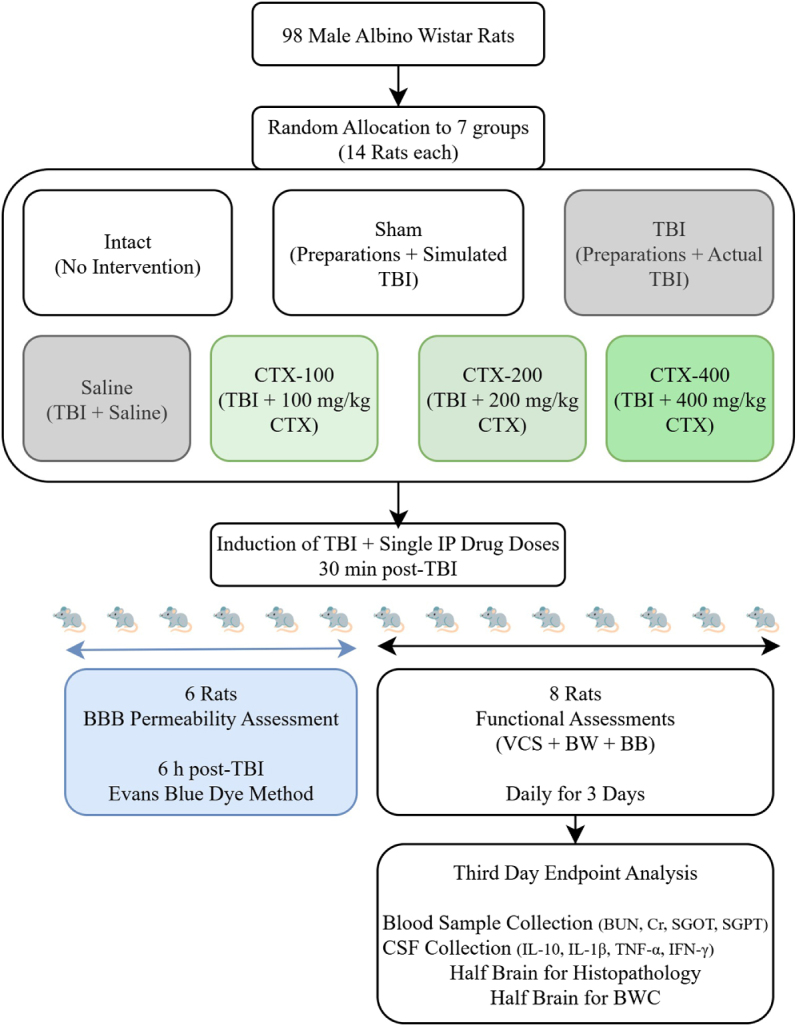
This figure summarizes the experimental design. The study utilized 98 male rats, allocated across seven groups. The Intact and Sham groups were not subjected to TBI or drug treatment. The TBI group received the actual trauma but no medication, while the Saline group received both the actual TBI and an IP injection of 0.9% saline as a placebo. Three therapeutic groups were administered a single IP injection of CTX at doses of 100, 200, or 400 mg/kg, 30 minutes post-TBI. From each group, six animals were euthanized six hours after the injury to assess BBB permeability using the Evans blue dye technique. The remaining eight rats in each group underwent daily neurobehavioral testing for three days. Upon completion of the tests, CSF and blood samples were collected from these animals for cytokine and systemic toxicity analysis before they were euthanized. The extracted brains were then divided; one hemisphere was reserved for histopathological examination, and the other for measuring cerebral edema. BBB, blood–brain barrier; CSF, cerebrospinal fluid; CTX, ceftriaxone; IP, intraperitoneal; TBI, traumatic brain injury.

A total of 98 male Albino Wistar rats (weighing 250–330 g) were housed under standard conditions. The work has been reported in line with the ARRIVE criteria[23]. They were kept in an air-conditioned room maintained at 22 ± 2 °C with a 12-hour light–dark cycle and provided unrestricted access to food and water. TBI was induced using the Marmarou free-fall technique[24]. Figure 1 demonstrates an overview of the methodology used in the present study. The rats were randomly assigned to the following groups (14 rats each, using a computer-generated random number sequence):

- Intact group: Rats that received no intervention.
- Sham group: Rats that underwent anesthesia, surgical opening, and falsely simulated TBI induction without actual injury.
- TBI group: These rats were anesthetized, surgically opened, and subjected to the actual TBI induction but received no medication or placebo.
- Saline group: Similar to the TBI group, these rats received the actual TBI in addition to an intraperitoneal (IP) injection of 0.9% normal saline (the solvent for CTX) 30 minutes post-TBI.
- CTX groups (TBI + 100, 200, or 400 mg/kg CTX): Rats received single IP injections of CTX at doses of 100 (CTX-100), 200 (CTX-200), or 400 (CTX-400) mg/kg, 30 min after TBI induction.

### Animal preparation and induction of diffuse TBI

The rats were anesthetized using IP injections of ketamine (100 mg/kg) and xylazine (5 mg/kg), intubated, and connected to a breathing pump to maintain controlled respiration and prevent hypoxia. Diffuse TBI was induced using the Marmarou method, which was thoroughly explained in our previous study[25]. A 3-mm-thick, 10-mm-diameter metallic disk was affixed to the skull between the bregma and lambda to distribute the impact force and prevent skull fractures. The rat was placed prone on a foam bed, and a 400-g steel cylinder was dropped from a 2-meter height to induce injury. Post-injury, the body temperature was maintained at 37 °C using a heating pad, and respiratory support was continued. After recovery, rats were housed individually.

**HIGHLIGHTS**

- Single-dose ceftriaxone (CTX) shows neuroprotection after severe traumatic brain injury (TBI).
- Optimal effects at 100 and 200 mg/kg, with a ceiling at 400 mg/kg.
- CTX improves function, reduces edema, and enhances blood–brain barrier integrity.
- Modulates post-TBI cytokines, raising IL-10 and lowering IL-1β, TNF-α, and INF-γ.
- Neuroprotection occurs without signs of systemic toxicity (liver or kidney).

The Marmarou weight-drop (impact-acceleration) model was selected for this study because it optimally replicates severe diffuse TBI, which is a common clinical presentation following high-impact events such as falls and motor vehicle accidents[26]. Furthermore, as a closed-head injury model, it more closely mimics the human condition of blunt-force trauma compared to models that require a craniectomy. While other models, such as the Controlled Cortical Impact, offer excellent reproducibility for focal injuries, the Marmarou model was chosen for its proven utility in studying the complex, brain-wide sequelae of severe TBI^[26–28]^.

### Assessment of BBB permeability

BBB permeability was assessed using 2% Evans blue dye (20 mg/kg, intravenous) in the brain tissue of six rats in each group. Six hours post-TBI, the rats were anesthetized with thiopental (50 mg/kg, IP), and Evans blue dye was injected via the jugular vein. After one hour, the circulatory system was flushed with 300 mL of heparinized saline through the left ventricle to remove the intravascular dye until a clean solution strolled out of the vein. The brains were then extracted, weighed, and homogenized. The homogenate was mixed with 14 cc acetone and 6 cc sodium sulfate, shaken for 24 h, and centrifuged at 2000 rpm for 10 min after adding 1 cc trichloroacetic acid to 1 cc of the supernatant solution^[29]^. Evans blue dye absorption was measured at 620 nm using a spectrophotometer (Pharmacia Biotech), and dye content was calculated using the following standardized formula:

Evans blue dye (µg) per g brain tissue = (13.24 × 20 × absorbance)/tissue weight (g)

### Assessment of neurological and motor outcomes

The remaining eight rats in each group underwent daily neurobehavioral assessments. Neurological function was evaluated using the Veterinary Coma Scale (VCS), which assesses motor, ocular, and respiratory actions[25]. Scores (ranging from 3 to 15) were recorded before trauma (Pre-TBI), on the day of trauma (D0), and on the first (D1), second (D2), and third (D3) days post-TBI, with lower scores indicating neurological deficits.

Vestibulomotor function was assessed using a recording camera with a beam task[25]. Prior to TBI, rats were trained on a 100-cm beam (4 cm wide) and a narrower 1.5-cm-wide beam (five times each). For the beam walk (BW) test, rats were tested on a 2.5-cm-wide, 100-cm-long beam, with traversal time and falls recorded over three attempts after TBI. For the beam balance (BB) test, the rats were placed on a 2-cm-wide beam, and their ability to balance for up to 60 s was scored every 12 s (maximum score of 5).

### CSF collection

At the end of the third day, initially, the animals were anesthetized using a combination of ketamine and xylazine. Their heads were then secured in a stereotaxic frame, and the hair on their necks was shaved. With the head positioned at a 45-degree angle, a polyethylene tube and a Hamilton syringe were employed to withdraw 200 µL of CSF from the Cisterna magna, situated between the occipital bone and the cervical atlas. CSF was meticulously collected to prevent any blood contamination, transferred into an Eppendorf tube, and promptly frozen in liquid nitrogen.

### CSF cytokine measurement

The concentrations of IL-10 and IL-1β in the CSF were measured using rat-specific interleukin 10 (IL-10) (MBS2020828, detection range: 7.8-500 pg/ml, sensitivity: < 3.5 pg/ml), IL-1β (MBS2023030, detection range: 15.6-1000 pg/ml, sensitivity: < 5.4 pg/ml), tumor necrosis factor alpha (TNF-α) (MBS175904, detection range: 15.6-1000 pg/ml, sensitivity: < 1 pg/ml), and interferon gamma (INF-γ) (MBS766197, detection range: 15.625-1000 pg/ml, sensitivity: 9.375 pg/ml) enzyme-linked immunosorbent assay (ELISA) kits obtained from MyBioSource, Inc. (San Diego, CA, USA), following the manufacturer’s protocols.

### Blood sample collection and systemic toxicity assessment

On the third day post-TBI, following CSF collection and prior to brain extraction, blood samples were collected for systemic toxicity analysis. Blood was drawn via cardiac puncture into sterile tubes and allowed to clot at room temperature. The samples were then centrifuged at 3000 rpm for 10 minutes to separate the serum. The collected serum was stored at −80°C until analysis. Liver and kidney functions were assessed by measuring serum levels of blood urea nitrogen (BUN) (MBS2611086, detection range: 0.312-20 mmol/L, sensitivity: up to 0.06 mmol/L), creatinine (MBS3809095, detection range: 0.5-8 mmol/L, sensitivity: 0.1 mmol/L), serum glutamic pyruvic transaminase (SGPT/ALT), and serum glutamic oxaloacetic transaminase (SGOT/AST) (both by GOT/GPT kit MBS160551, detection range: 2-700 U/L, sensitivity: 2-700 U/L) using rat-specific ELISA kits according to the manufacturer’s protocols.

### Histopathological evaluation and brain water content analysis

Finally, after CSF collection and induction of deep anesthesia, brains of these eight rats were rapidly extracted. Half of the brain was used for histopathological assessments, and the other half was used for BWC analysis.

First half was fixed in 10% formaldehyde buffer for 24 h. Tissues were processed, dehydrated, embedded in paraffin, sectioned at 5 µm thickness using an automatic microtome, and stained with hematoxylin–eosin (H&E). Then, blinded histopathology examined parameters such as degeneration, vacuolation, inflammation, edema, apoptosis, and neuronal death in the brain cortex under a light microscope. The BWC was determined on the other half of the brain. The tissues were weighed (wet weight), dried at 60–70 °C for 72 h, and reweighed (dry weight). The percentage brain water content was calculated using the dry-wet weight method[30].

### Statistical analysis

For normally distributed data (assessed by Shapiro-Wilk’s test), analysis of variance (ANOVA) was used to compare groups for statistical significance, followed by post hoc test Tukey HSD. The Kruskal–Wallis test was applied to non-normally distributed data followed by Dunn’s test for post hoc analysis. The area under the curve (AUC) was calculated using the ΔX × ([(Y1 + Y2)/2)] − baseline) formula. Data analysis was conducted using Prism 5 GraphPad software and presented as mean ± standard error of the mean (SEM) with a significance level of *P* < 0.05.

## Results

### Neurological and motor function

**Figure 2.**
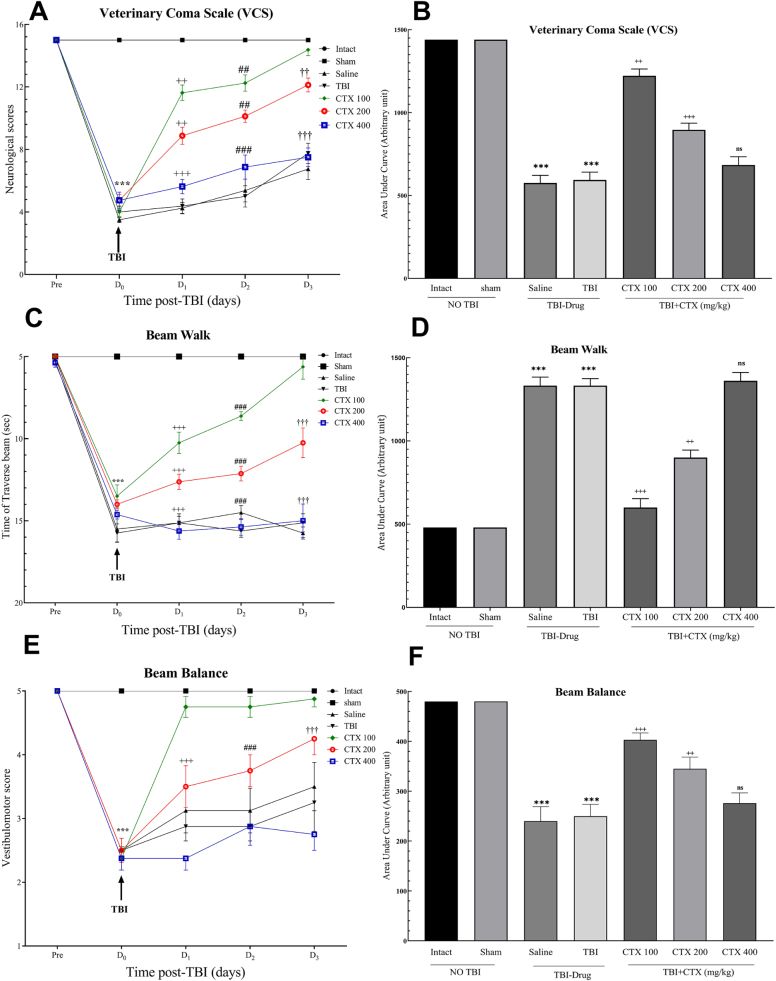
A–C: Each point shows the mean ± SEM. All neurological and motor function scores improved significantly after TBI in the CTX-100 and CTX-200 groups; however, this was not observed in the CTX-400 group. A: VCS scores, B: AUC of VCS score, C: BW time, D: AUC of BW, E: BB time, F: AUC of BB. AUC calculation also shows significant improvements in the CTX-100 and CTX-200 groups after TBI (* *P* < 0.05, ** *P* < 0.01, *** *P* < 0.001, ++ *P* < 0.01, +++ *P* < 0.001, ## *P* < 0.01, ### P <0 .001, †† *P* < 0.01, ††† P <0 .001, ns: not significant). AUC, area under the curve; BB, beam balance; BW, beam walk; CTX, ceftriaxone; SEM, standard error of the mean; TBI, traumatic brain injury; VCS, Veterinary Coma Scale.

Our findings demonstrated that VCS, BW, and BB scores were effectively reduced in all testing groups after TBI, while CTX administration at 100 mg/kg and 200 mg/kg dosages significantly improved the neurological status and motor function of the rats on D_3_ with the 100 mg/kg dose showing the greatest improvement. In contrast, no significant improvement was observed in the CTX-400 group. Similarly, the AUC calculation showed improvements with CTX-100 and CTX-200, but no significant results with CTX-400 administration (Fig. 2).

### Cerebral edema

**Figure 3.**
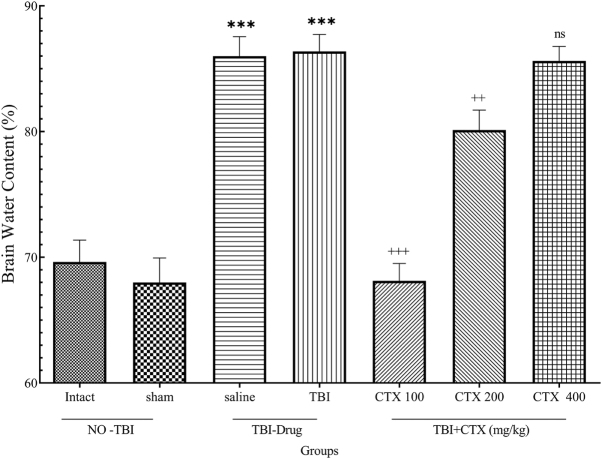
Brain water content (+++ *P*< 0.05, ++ *P*< 0.01, *** *P*< 0.001, ns: not significant).

Figure 3 shows the percentages of BWC in the study groups. The results showed that 100 mg/kg CTX prevented water accumulation in the brain. Additionally, 200 mg/kg CTX resulted in a reduction in brain edema after TBI; however, 400 mg/kg CTX demonstrated no significant effects on BWC after trauma.

### BBB permeability

**Figure 4.**
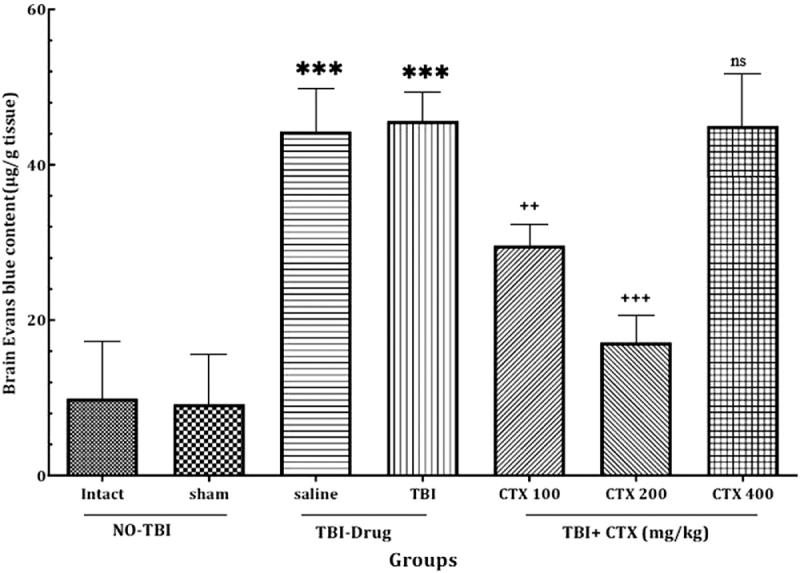
Brain Evans blue dye content for BBB permeability evaluation (+++ *P*<0.05, ++ *P*<0.01, *** *P*<0.001, ns: not significant). BBB, blood–brain barrier.

BBB permeability was measured using Evans blue dye content in the brain 24 h post-trauma. As shown in Figure 4, TBI induction resulted in a significant increase in Evans blue dye content in the brain. However, administration of 100 mg/kg and 200 mg/kg CTX caused a significant reduction in the Evans blue dye content in the brain, with the CTX-200 group showing better improvements. Remarkably, the CTX-400 group demonstrated no significant change in BBB permeability compared with the TBI and saline groups.

### CSF cytokine measurements

**Figure 5.**
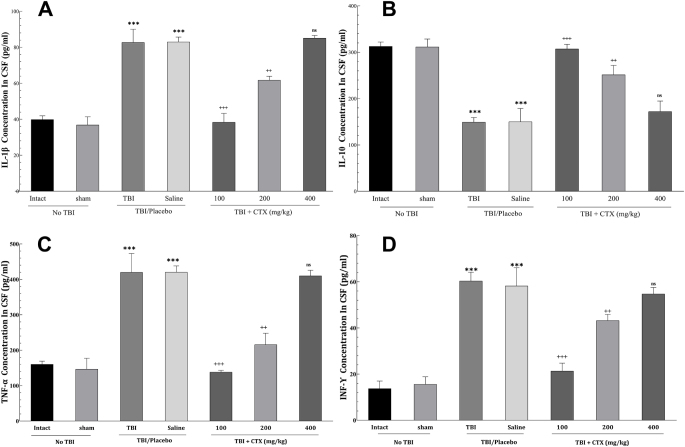
Concentration of interleukins in the CSF. A: Concentration of IL-1β in the CSF; B: Concentration of IL-10 in the CSF; C: Concentration of TNF-α in the CSF; and D: Concentration of IFN-Ɣ in the CSF (*** *P* < 0.001, ++ *P* < 0.01, +++ *P* < 0.001, ns: not significant). CSF, cerebrospinal fluid; IFN-γ, interferon gamma; IL-1β, interleukin‑1 beta; IL-10, interleukin‑10; TNF-α, tumor necrosis factor alpha.

The concentration of IL-1β, TNF-α, and INF-Ɣ were notably lower in the CTX-100 and CTX-200 groups than in the TBI and saline groups, whereas the concentration of IL-10 was significantly higher in these groups compared to the controls. However, no significant changes in the levels of any of the cytokines were observed in the CTX-400 group (Fig. 5).

### Histopathological alterations

**Figure 6.**
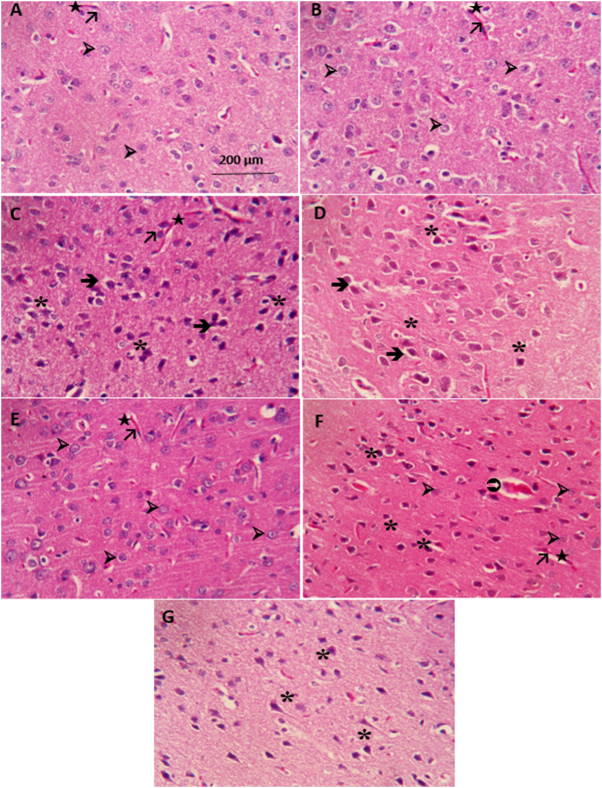
A and B: Microscopic image of the cerebral cortex of adult rats in the Intact and Sham groups. Normal neurons with large central vesicular nuclei and neuroglia are depicted. C and D: Microscopic image of the cerebral cortex of adult rats in the TBI and TBI + Saline groups. Degenerated neurons with pyknotic and intensely stained nuclei are observed, accompanied by perineuronal edema, vascular congestion, and perivascular edema. E: Microscopic image of the cerebral cortex of adult rats in the CTX-100 group. Normal neurons with large central vesicular nuclei and neuroglia are observed. F: Microscopic image of the cerebral cortex of adult rats in the CTX-200 group. Normal neurons are seen alongside degenerated neurons with pyknotic and intensely stained nuclei, accompanied by perineuronal edema. G: Microscopic image of the cerebral cortex of adult rats in the CTX-400 group. Degenerated neurons with pyknotic and intensely stained nuclei are present, along with perineuronal edema, vascular congestion, and perivascular edema. All images are magnified at × 400 and stained with H&E (➢: normal neuron, _*_: degenerated neuron, ➜: edematous neuron, ➲:swollen astrocyte, ★: blood vessel, ↗: endothelial cell). CTX, ceftriaxone; H&E, hematoxylin–eosin; TBI, traumatic brain injury.

The histopathological alterations observed in various experimental groups are shown in Figure 6. The intact and sham groups displayed a typical histological structure in the cerebral cortex, characterized by basophilic, euchromatic, and oval-shaped somas. In contrast, the TBI and saline groups showed notable histopathological deviations, including neurons with edematous and irregular shapes as well as darkly stained nuclei. Additionally, these groups exhibited perivascular edema, necrotic neurons, endothelial cell swelling, and vascular congestion. In the CTX-100 group, a significant decrease in the number of degenerated and edematous neurons was observed, suggesting a possible neuroprotective effect. The CTX-200 group primarily displayed normal neuronal morphology, featuring euchromatic nuclei and distinct nucleoli. Moreover, the endothelial cells, blood vessels, and astrocytes in these groups maintained a normal structure, similar to that observed in the saline group. In contrast, the CTX-400 group presented severe pathological alterations, closely resembling those observed in the TBI and Saline groups.

### Systemic toxicity assessment

**Figure 7.**
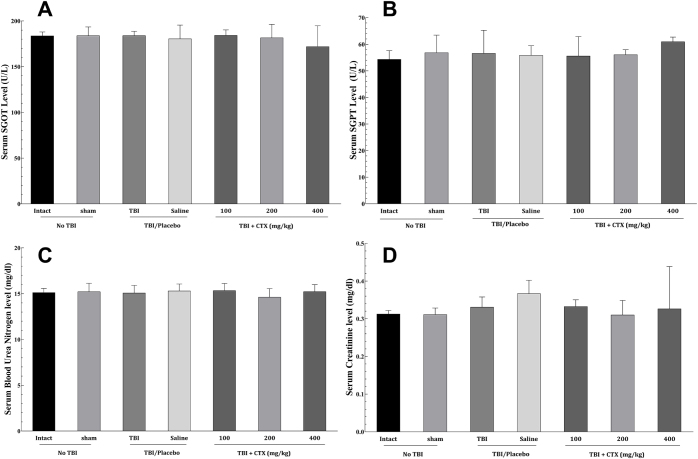
Assessment of systemic toxicity following CTX administration. Serum levels of (A) SGOT (AST) and (B) SGPT (ALT) as markers of hepatotoxicity, and (C) Blood Urea Nitrogen (BUN) and (D) Creatinine as markers of nephrotoxicity, were measured on the third day post-TBI. Data are presented as mean ± SEM. No significant differences were observed in any of the CTX-treated groups (100, 200, or 400 mg/kg) compared to the Intact, Sham, or Saline groups, indicating the absence of significant liver or kidney damage at the tested doses. Statistical significance was assessed by one-way ANOVA followed by post-hoc tests. ALT, alanine aminotransferase; ANOVA, analysis of variance; AST, aspartate aminotransferase; BUN, blood urea nitrogen; CTX, ceftriaxone; SEM, standard error of the mean; SGOT, serum glutamic oxaloacetic transaminase; SGPT, serum glutamic pyruvic transaminase; TBI, traumatic brain injury.

To evaluate the potential systemic toxicity of CTX, serum markers of liver (SGOT, SGPT) and kidney (BUN, creatinine) function were analyzed. As shown in Figure 7, there were no significant increases in the levels of SGOT, SGPT, BUN, or creatinine in any of the CTX-treated groups (100, 200, or 400 mg/kg) compared to the intact, sham, or saline group. This indicates that a single administration of CTX at the doses tested did not induce significant hepatotoxicity or nephrotoxicity in this model.

## Discussion

In this study, we investigated the neuroprotective effects of CTX on brain edema, BBB permeability, and neurological and motor functions in male rats following severe TBI. The findings confirmed that CTX significantly inhibited water accumulation in the brain, improved BBB function, enhanced neurological recovery as demonstrated by the VCS, BW, and BB tests, improved histological changes, reduced IL-1β, TNF-α, and IFN- γ, and increased IL-10 levels after TBI.

Hameed *et al*[19] provided valuable insights into the molecular mechanisms through which CTX exerts its neuroprotective effects. TBI leads to a significant reduction in the expression of glutamate transporter-1 (GLT-1), the primary glutamate transporter responsible for clearing synaptic glutamate. This reduction coupled with increased glutamate release from damaged neurons results in elevated extracellular glutamate levels, leading to glutamate excitotoxicity. Excitotoxicity disproportionately affects GABAergic and parvalbumin-positive (PVALB +) inhibitory interneurons, which are crucial for maintaining cortical inhibitory tone. The loss of these interneurons contributes to a progressive excitatory-inhibitory imbalance associated with chronic posttraumatic symptoms. Hameed *et al*[19], Goodrich *et al*[31], and Cui *et al*[6] demonstrated that CTX significantly attenuates the loss of GLT-1. This is particularly relevant to our study, as excitotoxicity is a key contributor to neuronal damage, BBB disruption, and brain edema following TBI; therefore, by reducing glutamate levels, CTX may help maintain BBB integrity and reduce edema. While not measured directly here, the primary mechanism of CTX neuroprotection could be attributed to the upregulation of the astrocytic glutamate transporter, GLT-1. Previous research has shown that CTX pre-treatment enhances both GLT-1 mRNA and protein expression[32]. Furthermore, the critical role of GLT-1 in mediating these effects could be confirmed by studies demonstrating that the selective GLT-1 antagonist dihydrokainate reverses CTX-induced neuroprotection and anti-nociception[33].

CTX treatment also preserved intracortical inhibition, as measured by long-interval paired-pulse transcranial magnetic stimulation with mechanomyography (LI-ppTMS-MMG)[19]. This suggests that CTX helps to maintain GABAergic inhibitory tone, which is otherwise progressively lost after TBI. In addition, the preservation of PVALB + inhibitory interneurons by CTX, as shown by Hameed *et al*[19], could explain the improvements in neurological and motor function in our study. The loss of these interneurons is associated with impaired cortical inhibition and motor deficits; therefore, their preservation may have contributed to the recovery of motor function observed in our subjects.

Although not directly addressed in the study by Hameed *et al*[19], the reduction in excitotoxicity and preservation of inhibitory interneurons may also mitigate secondary injury mechanisms such as oxidative stress and inflammation, which exacerbate BBB disruption and brain edema. Increased extracellular glutamate after trauma can cause excess intracellular calcium, which may lead to the production of reactive oxygen species^[34,35]^. Wei *et al*[36] investigated the neuroprotective effects of CTX after TBI through inflammatory responses and found that TBI led to a significant increase in proinflammatory cytokines, including IL-1β, IFN-γ, and TNF-α. However, CTX treatment significantly reduced the levels of these cytokines, suggesting an anti-inflammatory effect of CTX. In the present study, we found that CTX treatment led to a reduction in IL-1β and an increase in IL-10 concentration. The combined effects of reduced excitotoxicity and inflammation likely contribute to the overall neuroprotective benefits of CTX, including improved cognitive function and reduced brain edema.

Cui *et al*[6] investigated the neuroprotective effects of CTX in a rat model of TBI. Similar to our findings, they showed that CTX treatment (200 mg/kg/day for 5 days) significantly reduced brain edema compared with the untreated TBI group, suggesting that CTX has a protective effect against pathological cerebral edema after TBI. Similarly, Wei *et al*[36] showed that TBI induced significant cerebral edema, which was attenuated by CTX treatment (200 mg/kg). However, our results demonstrated that although both 100 and 200 mg/kg doses significantly reduced brain edema, the 100 mg/kg dose of CTX exerted a greater reduction in BWC than the 200 mg/kg dose. While both doses were significantly effective compared to the TBI control, this inverse relationship for edema reduction suggests a complex pharmacological interaction. It is plausible that at the higher dose of 200 mg/kg, other, more subtle biological responses are engaged. These could include a mild, sub-clinical shift in neuroinflammatory signaling or off-target effects that partially counterbalance the potent anti-edema effects seen at 100 mg/kg. This observation underscores that the dose–response relationship for CTX’s neuroprotection may be system-specific and that a single, optimal dose for all outcome measures may not exist. Future studies measuring aquaporin-4 expression across these doses could provide mechanistic clarity for this particular finding.

Cui *et al*[6] also showed that TBI causes significant deficits in spatial learning and memory, as measured by the Morris water maze (MWM) test. However, CTX treatment improved cognitive function and reduced escape latency at 3 and 5 d post-TBI. This indicates that CTX not only mitigates physical damage but also enhances functional recovery after TBI. Similarly, Wei *et al*[36] showed that CTX significantly improved cognitive function, reducing the time of reaction and error number in the Y-maze test at 7 days post-injury. Although we did not conduct the MWM test in the present study, our results also showed improvements in neurological and motor function after CTX treatment.

Cui *et al*[6] showed that CTX treatment suppressed the increase in LC3 II expression (a marker of neuronal autophagy) post-TBI. This suggests that CTX protects neurons by reducing excessive autophagy, which is associated with neuronal death after TBI. In addition, in a study by Goodrich *et al*[31], CTX treatment reduced glial fibrillary acidic protein expression by 43% (which increased after TBI), indicating a reduction in reactive astrogliosis. This may contribute to the neuroprotective effects of CTX after TBI because excessive gliosis is associated with impaired neuronal function and epileptogenesis.

The observed loss of therapeutic efficacy at the highest dose (400 mg/kg) delineates a classic inverted-U or U-shaped dose–response curve, a phenomenon common in neuropharmacology where both insufficient and excessive dosing can be ineffective or detrimental. While systemic toxicity was ruled out by our serum analyses, the failure of CTX-400 is likely attributable to mechanisms that countervail its primary benefits. First, at supraphysiological concentrations, CTX may exhibit off-target effects. Notably, some beta-lactam antibiotics have been demonstrated to act as weak antagonists at GABA-A receptors[22]. This GABAergic antagonism could induce neuronal disinhibition and increase network excitability, thereby directly opposing the anti-excitotoxic effect achieved through GLT-1 upregulation at lower, optimal doses[36].

### Clinical translation

The clinical translation of the findings from this study highlights the potential of CTX as a promising neuroprotective agent for TBI treatment. Collectively, these studies demonstrated that CTX, a well-tolerated β-lactam antibiotic with excellent BBB penetration, can mitigate key secondary injury mechanisms following TBI, including glutamate excitotoxicity, inflammation, and cerebral edema. By upregulating GLT-1, CTX enhances glutamate clearance and reduces excitotoxic neuronal damage, while its anti-inflammatory properties help suppress proinflammatory cytokines, such as IL-1β, IFN-γ, and TNF-α, which exacerbate secondary injury. Additionally, CTX has been shown to improve cognitive function, reduce post-traumatic seizures, and attenuate brain edema, all of which are critical for improving the outcomes in patients with TBI. The safety profile and pharmacokinetics of CTX, already well established in clinical practice for treating bacterial infections, further support its feasibility for rapid translation into clinical trials for TBI. The present study used relatively high doses of CTX (100, 200, and 400 mg/kg), which were higher than the typical clinical doses. However, even normal doses of CTX used for bacterial infection (1–2 g/day in adults or 50 mg/kg in pediatric patients) may be sufficient to achieve neuroprotective effects. Thus, future studies are needed to optimize the dosing regimens, determine the therapeutic window, and confirm the long-term benefits of CTX in patients with TBI.

The therapeutic window for neuroprotection is critical. Our study supports the strategy of early administration to mitigate secondary injury. We administered CTX 30 minutes post-TBI in our rat model to target the initial surge in excitotoxicity and inflammation. This aligns with the pathophysiological understanding that secondary injury mechanisms like glutamate excitotoxicity begin within minutes to hours after the initial trauma^[10,37]^. Translating this to humans, the goal would be to administer CTX as soon as possible after resuscitation and stabilization, likely in the pre-hospital setting or emergency department, to intercept these damaging cascades early.

A significant advantage of CTX is its potential for seamless integration into existing TBI treatment protocols. The current standard of care for severe TBI focuses on preventing secondary injury by maintaining hemodynamic stability, monitoring and treating intracranial pressure (ICP), and ensuring adequate cerebral perfusion pressure ^[37–39]^. CTX, proposed as an adjunctive therapy, would complement these measures by targeting the molecular drivers of injury, such as glutamate excitotoxicity and neuroinflammation, which are not directly addressed by current standard management. Furthermore, the feasibility of this approach is supported by recent clinical evidence. A large, randomized controlled trial (the PROPHY-VAP trial) demonstrated that a single, early dose of CTX is safe and effective in preventing ventilator-associated pneumonia in comatose patients with acute brain injury, leading to improved outcomes such as shorter ICU stays and reduced mortality[40]. This evidence powerfully underscores the practicality and safety of incorporating a single CTX dose into the initial management bundle for severe TBI patients. Collectively, these findings underscore the potential of CTX as a multifaceted therapeutic intervention to address the complex pathophysiology of TBI and improve patient outcomes.

### Clinical feasibility

The clinical translation of our dosing regimen is highly feasible. Using standard body surface area conversion methods[41], the effective rat doses of 100 and 200 mg/kg translate to human-equivalent doses (HEDs) of approximately 1.1 g (~16 mg/kg) and 2.2 g (~32 mg/kg) for a 70 kg adult, respectively. These HEDs are within the well-established and routinely used clinical range of 1–2 g per day for severe infections like meningitis, with the 2.2 g dose being at the higher end but well within the maximum safe daily dose of 4 g. The single-dose administration in our study further aligns with prophylactic or acute intervention protocols, minimizing risks associated with prolonged antibiotic use. Importantly, the lack of further efficacy at the 400 mg/kg dose (HED: ~ 65 mg/kg, ~ 4.6 g for a 70 kg adult), which exceeds the maximum recommended dose, indicates a therapeutic ceiling. This is a positive finding for translation, as it confirms that lower, clinically safe, and feasible doses are effective for neuroprotection.

### Future research direction

This study was exclusively conducted on male rats. This choice was made to control for the well-documented neuroprotective and immunomodulatory effects of female sex hormones (e.g., estrogen and progesterone), whose fluctuating levels across the estrous cycle introduce significant variability in neuroinflammatory and excitotoxic pathways. While this allowed for a clearer initial characterization of the CTX dose-response curve, it limits the generalizability of our findings. Future studies are unequivocally needed to investigate the efficacy of CTX in female subjects. These studies should directly compare males and females, and within females, should account for hormonal status by either using ovariectomized models with hormonal replacement or by tracking the estrous cycle to determine if CTX’s neuroprotective effects are phase dependent.

## Conclusion

Our results demonstrated that administering CTX at doses of 100 and 200 mg/kg significantly improved neurologic and motor outcomes, reduced cerebral edema, enhanced BBB integrity, and improved the inflammatory response in TBI-induced rats with no signs of systemic toxicity.

## Data Availability

The data are available upon reasonable request from the corresponding author.

## Declaration of AI use

During the preparation of this work, the authors used “Grammarly” in order to improve the language proficiency of the manuscript on 12 October 2025. Authors confirm that all inputs were de-identified and compliant with GDPR/HIPAA. After using this tool/service, the supervising authors (Dr. Ali Siahposht-Khachaki and Dr. Erfan Ghadirzadeh) reviewed and edited the content as needed. We confirm that all authors reviewed the final content and take full responsibility for the content of the publication. Additionally, authors disclose no conflicts of interest or financial ties to AI vendors.
